# Development of a model to guide the decision for radiotherapy in deep-inspiration breath-hold in patients with left-sided breast cancer

**DOI:** 10.1007/s00066-025-02438-4

**Published:** 2025-07-29

**Authors:** Annett Linge, Carina Ketzmerick, Kristin Gurtner, Cordelia Hoinkis, Steffen Löck, Mechthild Krause

**Affiliations:** 1 Department of Radiotherapy and Radiation Oncology, Faculty of Medicine and University Hospital Carl Gustav Carus, Technische Universität Dresden, Dresden, Germany; 2 OncoRay – National Center for Radiation Research in Oncology, Faculty of Medicine and University Hospital Carl Gustav Carus, Technische Universität Dresden, Helmholtz-Zentrum Dresden - Rossendorf, Dresden, Germany; 3 German Cancer Research Center (DKFZ), Heidelberg, Germany, and German Cancer Consortium (DKTK), partner site Dresden, Germany; 4 National Center for Tumor Diseases (NCT), Partner Site Dresden, Germany: German Cancer Research Center (DKFZ), Heidelberg, Germany, Faculty of Medicine and University Hospital Carl Gustav Carus, Technische Universität Dresden, Dresden, Germany, and Helmholtz Association / Helmholtz-Zentrum Dresden - Rossendorf (HZDR), Dresden, Germany; 5 Helmholtz-Zentrum Dresden-Rossendorf, Institute of Radiooncology – OncoRay, Dresden, Germany

**Keywords:** Cardiac toxicity, DIBH, Heart-sparing techniques, Gating, Irradiation

## Abstract

Patients with left-sided breast cancer receiving radiotherapy are at risk of developing chronic cardiac toxicities. Cardiac dose-sparing techniques such as deep-inspiration breath-hold (DIBH) can reduce this risk. In this study, a model has been developed including tumor localization, left lung volume, and the distance between the heart and medial chest wall as well as age and Eastern Cooperative Oncology Group (ECOG) performance status, which may help to guide the decision for or against DIBH after planning computed tomography (CT) in free breathing directly before initiation of radiotherapy planning.

## Introduction

Radiotherapy of breast cancer, as part of a multidisciplinary approach, has been shown to reduce the risk of local recurrences and is therefore recommended in national and international guidelines depending on tumor stage and the extent of surgery.

Cardiac toxicity has been identified as late toxicity after irradiation, which mostly appears 10–15 years after irradiation [1] and can lead to cardiac infarction, angina pectoris, and cardiac insufficiency as well as arrythmia, pericarditis, cardiomyopathy, and other sequalae [2, 3]. Most importantly, higher morbidity and mortality have been linked to left-sided breast cancer [4]. This is mainly due to the small distance between the chest wall and the heart with its critical substructures including the coronary arteries [5, 6]. In addition, breast cancer patients may also receive (neo)adjuvant systemic therapy, which can itself be cardiotoxic and/or lead to additive cardiac toxicity when combined with radiotherapy [7, 8].

In the past two decades, advanced radiotherapy techniques have led to reduced heart doses [9]; however, these alone are usually not sufficiently dose sparing [10]. One method to further decrease the dose to the heart is breast irradiation in deep-inspiration breath-hold (DIBH) [11], and its robustness against intrafractional motion has recently been demonstrated [12]. Irradiation in DIBH can lead to a significant reduction of the dose to the heart and coronary vessels as well as of the dose to the lungs, while the dose coverage of the target volume remains unaffected [13–16], as recently been confirmed in the SAVE-HEART study, a large prospective clinical trial [17]. Deep-inspiration breath-hold has been recommended as a heart-spearing technique in combination with modern radiotherapy techniques by the expert panel of the German Society of Radiation Oncology e. V. [10]. Irradiation in DIBH, when combined with volumetric modulated arc therapy instead of three-dimensional conformal radiotherapy has also been shown to further decrease high doses to organs at risk; however, the volume of normal tissue receiving low doses increases [18].

Patients’ ability to perform DIBH depends on physiological factors such as lung capacity and pulmonary function as well as on their ability to correctly perform this method from the technical aspect (constant breath-hold and thoracic breathing) [19, 20]. The DIBH irradiation itself is more time consuming in daily practice and may also require some training before the planning computed tomography (CT) scan. A recent study showed that the planning CT can usually be scheduled directly after DIBH training for the same day, as a time interval between DIBH training and the planning CT did not lower the dose to organs at risk (OARs) [21]. Taken together, indicators are needed to identify patients who are both able to perform the DIBH technique and who will likely receive heart-sparing doses.

Therefore, in this study, we retrospectively explored parameters in patients with left-sided breast cancer, which may have led to the decision regarding whether the patient received breast irradiation in DIBH or in free breathing and developed a model that might help to identify these patients after planning CT in free breathing before initiation of radiotherapy treatment planning.

## Materials and methods

### Patient cohort

**Fig. 1:**
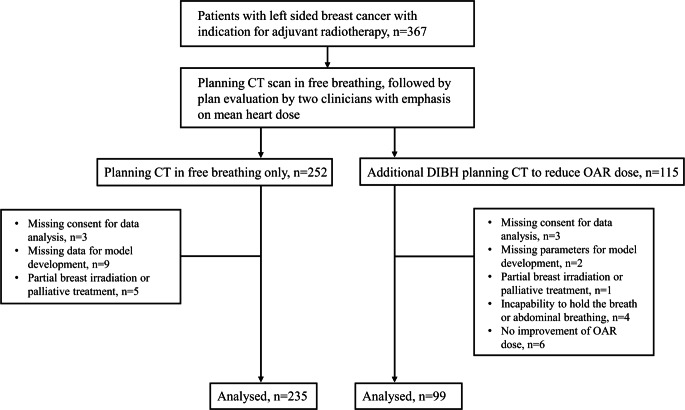
Flowchart of patient selection. *CT* computed tomography, *OAR* organ at risk

In this retrospective study, patients with left-sided breast cancer or ductal carcinoma in situ (DCIS) who received adjuvant radiotherapy according to national guidelines were included (Fig. 1). All patients were treated at the Department of Radiotherapy and Radiation Oncology at the University Hospital Carl Gustav Carus in Dresden (Germany) and received a planning CT scan with 2 mm slice thickness in free breathing and, if deemed eligible according to the treating physician, in DIBH. In the DIBH subgroup, 115 consecutive patients were included (treated from 2014 to 2019) who had received two planning CT scans—one in free breathing and one in DIBH. Enrolment in the DIBH group was independent of the final decision for irradiation in DIBH or free breathing. In the control group with free breathing, 252 consecutively treated patients were enrolled (treated from 2016 to 2018). For the patients in the control group, the DIBH technique was clinically not considered, since the median dose to the heart was within the constraints accepted at the time (Dmean up to 5 Gy) according to our clinical standard operating procedure (SOP). Next, patients were excluded if at least one of the following criteria was met: missing patient consent to use of their patient-related data for scientific analysis, missing parameters for model development, partial breast irradiation, or palliative-intent radiotherapy. We also had to exclude patients with lymph node irradiation other than in axilla level I or II, since DIBH was not yet established in our center at this time for irradiation with more than one isocenter. Patients were also excluded if the DIBH plan could not be applied, i.e., if the patient was unable to hold their breath or in the case of abdominal breathing. The DIBH plan had to be superior to the plan in free breathing based on the constraints for the organs at risk (e.g., heart, lung, left anterior descending artery [LAD]) as evaluated by two radiation oncologists. This led to the exclusion of 24 patients in the DIBH subgroup and of 72 patients in the control subgroup with free breathing, resulting in 91 and 180 patients, respectively.

The study has been approved by the local ethics committee and has therefore been performed in accordance with the ethical standards laid down in the 1964 Declaration of Helsinki and its later amendments.

### Parameters for model development

**Fig. 2:**
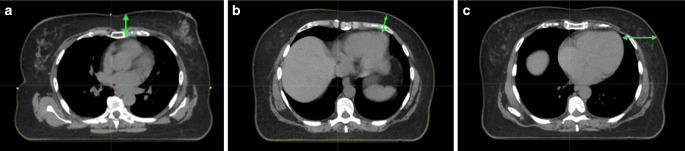
Axial CT slices with measurements (green arrows) of the **a** medial distance between the heart and the medial chest wall, **b** lateral distance between the heart and the lateral chest wall, and **c** mediolateral distance between the outer heart contour and the chest wall between the medial and lateral distance

For model development, the following parameters that are potentially available before planning CT were considered: age, Eastern Cooperative Oncology Group (ECOG) performance status, previous irradiation, systemic therapy, and tumor localization. Patient characteristics are presented in Table 1. In addition, parameters obtained directly after planning CT in free breathing were extracted, including the heart and lung volumes and the distance between the heart and the chest wall. The axial CT slice with the lowest distance was selected. The medial distance included the space between the medial chest wall and the heart (vertically), the lateral distance between the heart and the chest wall (horizontally), and the mediolateral distance between the outer heart contour and the chest wall between the medial and lateral distance (Fig. 2).

**Table 1:** Comparison of categorical and metric parameters of patients treated in deep-inspiration breath-hold (DIBH) or free breathing (control group)

Parameter		DIBH group	Control group	
*–*	*Categories*	*Number (fraction)*	*Number (fraction)*	*p-value*
ECOG performance status	0	87 (96%)	129 (72%)	–
	1–2	4 (4%)	51 (28%)	< 0.001*
Previous irradiation	No	90 (99%)	166 (92%)	–
	Yes	1 (1%)	14 (8%)	0.023*
Anthracyclines	No	47 (52%)	126 (70%)	–
	Yes	44 (48%)	54 (30%)	0.003*
Antibodies	No	76 (84%)	161 (89%)	–
	Yes	15 (16%)	19 (11%)	0.16
Chemotherapy	No	46 (51%)	117 (65%)	–
	Yes	45 (49%)	63 (35%)	0.022*
Localization	Other	59 (65%)	147 (82%)	–
	Upper/lower inner quadrant of the left breast	32 (35%)	33 (18%)	0.002*
Lymph node region irradiation (axilla levels I + II)	No	82 (90%)	154 (86%)	–
	Yes	9 (10%)	26 (14%)	0.29
*–*	*Unit*	*Median (range)*	*Median (range)*	*p-value*
Age	Years	51 (29–72)	65 (26–86)	< 0.001*
Total lung volume	cm^3^	2492 (1701–4038)	2692 (1717–4736)	0.018*
Left lung volume	cm^3^	1095 (646–1855)	1183 (779–2179)	0.027*
Heart volume	cm^3^	568 (375–819)	562 (381–868)	0.88
Distance heart–medial chest wall	cm	3.1 (1.4–5.1)	3.6 (1.4–7.1)	< 0.001*
Distance heart–lateral chest wall	cm	5.4 (2.7–10.7)	6.4 (3.0–15.5)	0.002*
Distance heart–mediolateral chest wall	cm	3.7 (1.8–7.5)	4.0 (1.6–8.6)	0.098

*ECOG* Eastern Cooperative Oncology Group, *DIBH *deep-inspiration breath-hold

*Statistically significant *p*-value

### Statistical analyses and model development

To assess differences in clinical and geometric characteristics between patients treated with and without DIBH, the Mann–Whitney U test was applied for metric variables, while the chi-squared test was applied for categorical variables. Considered variables are given in Table 1. Significant variables were subsequently included in a forward-selection logistic regression model to predict the use of DIBH. Thresholds for variable inclusion and exclusion were set to *p* = 0.05. The area under the receiver operating characteristics curve (AUC) was applied to assess model performance. The Youden index was used as a cutoff for the predicted probability for DIBH application. Sensitivity and specificity of the model were calculated for this cutoff. In addition, mean and maximum doses to the heart as well as the maximum dose to the LAD were available. For all analyses, a significance level of 0.05 was applied, and two-sided tests were used. Analyses were performed with SPSS 27 (IBM Corp., Armonk, NY, USA).

## Results

### Univariable comparisons between the DIBH and control groups

For the DIBH group, tumor localization was more often in the inner quadrants of the breast (35% in the DIBH group compared to 18% in the control group; *p* = 0.002) compared to other localizations. Patients in the DIBH group had a significantly higher ECOG performance status (0 vs. 1/2; *p* < 0.001), and only one patient of the DIBH group had previous/simultaneous irradiation of the right breast compared to 14 in the control arm (*p* = 0.023). Patients treated in DIBH more often received anthracyclines (*p* = 0.003) and/or chemotherapy (*p* = 0.002) compared to those treated in free breathing. In addition, a significant difference between the groups was observed for age, total lung volume, left lung volume, medial distance, and lateral distance between the chest wall and the heart (Table 1).

### Model development

The variables identified to be significantly different between the two patient groups (Table 1) were entered into forward-selection logistic regression to predict the use of DIBH. The following parameters were selected: ECOG performance status, tumor localization, age, volume of the left lung, and medial distance between the heart and the chest wall. Deep-inspiration breath-hold was preferably applied for patients with high ECOG performance status, tumor localization in the upper or lower inner quadrant of the left breast, young age, small lung volume, and small distance between the heart and the chest wall. The model showed an AUC of 0.83 (95% confidence interval: 0.79–0.88). With this model, the probability for DIBH application is estimated as: 1/{1 + exp[−(8.484 − 1.422 × ECOG + 0.762 × localization − 0.0756 × age − 0.00257 × left lung volume − 0.535 × distance heart–medial chest wall)]}. At the optimal cutoff of 0.25, a sensitivity of 0.90 and a specificity of 0.65 were observed. The final model parameters are given in Table 2.

**Table 2:** Final model parameters estimated in forward-selection logistic regression predicting the use of deep-inspiration breath-hold (DIBH)

Parameter	Coefficient	Odds ratio	95% confidence interval	*p*-value
ECOG performance status (0 [b] vs. 1–2)	−1.422	0.24	0.07–0.79	0.018*
Localization (other [b] vs. upper/lower inner quadrant of the left breast)	0.762	2.14	1.07–4.30	0.032*
Age (years)	−0.0756	0.93	0.90–0.96	< 0.001*
Left lung volume (cm^3^)	−0.00257	0.997	0.996–0.999	< 0.001*
Distance heart-medial chest wall (cm)	−0.535	0.59	0.39–0.88	0.010*
Constant	8.484	4839	–	< 0.001*

*ECOG* Eastern Performance Oncology Group, *DIBH* deep-inspiration breath-hold, *[b]* baseline

*Statistically significant *p-*value

## Discussion

In this study, parameters were explored that could potentially guide the clinical decision regarding whether application of the DIBH technique is likely to reduce the dose to the heart in patients with left-sided breast cancer, thus potentially reducing the risk of development of chronic heart disease in the future. Moreover, we developed a model that could help to identify these patients before initiation of radiotherapy treatment planning.

In clinical routine, any benefit from DIBH irradiation is evaluated during the radiotherapy plan comparison between the plans in free breathing and in DIBH [20, 22], which is time consuming with respect to the whole planning process. The model developed here does not only consider physical parameters in terms of constraints of organs at risk but also clinical parameters of the individual patients. Parameters with a direct influence on DIBH irradiation are tumor localization, the volume of the left lung, and the medial distance from the chest wall to the heart, while age and ECOG performance status are factors that may influence the capability of the patient to perform DIBH. In addition, the DIBH technique also requires patients to constantly hold their breath and avoid abdominal breathing.

This analysis showed that fewer patients received DIBH with increasing age. It must be considered that some treatment-related heart toxicities only develop after 10 to 15 years [1]. In patients with a lower life expectancy, irradiation in free breathing may therefore lead to reduction of local recurrences and tumor-dependent overall survival without increasing heart toxicity.

Patients with tumor localization in inner quadrants were more often assigned to the DIBH subgroup, which is likely due to the close distance between the chest wall and the heart and, thus, to a higher heart dose. This is in line with Bouchardy et al., who found a higher cardiac mortality in patients with lesions within the inner quadrants of the left breast [23]. Moreover, patients with smaller lung volumes are more likely to be assigned to the DIBH subgroup. Here, less lung volume between the chest wall and the heart leads to higher doses to the heart. This is possibly why the volume of the left lung was considered instead of the total lung volume in the model. However, both parameters were strongly correlated.

It is well known that the individual patient anatomy influences the heart dose and consequently cardiac toxicity [24, 25]. In several studies, potential anatomical parameters were analyzed for predicting the mean heart dose before initiation of treatment planning, e.g., in [22, 26, 27]. Lee et al. showed an association with the number of CT slices in which the heart was close to the chest wall [26], whereas Hiatt et al. measured the contact length between the heart and the anterior chest wall [27]. In Cao et al., a model was developed to predict the reduction of heart and lung doses in DIBH irradiation [22], with the lateral heart-to-chest distance/cardiac contact distances in parasagittal CT planes as strong predictors. Here, the medial distance between the outer chest wall was selected in the model, although there was a strong correlation between both the medial and the lateral distance (Spearman correlation 0.57), suggesting that both are indicative of benefits from DIBH irradiation. Previous or parallel irradiation of the contralateral breast was not selected by the model. Moreover, the application of systemic therapy seemed to be less important for assigning patients to the subgroups.

Overall, DIBH irradiation led to significantly lower mean (mean ± standard deviation DIBH: 2.0 ± 1.0 Gy vs. free breathing: 3.4 ± 1.6 Gy; *p* < 0.001) and maximum (30.8 ± 13.9 Gy vs. 40.6 ± 10.7 Gy; *p* < 0.001) doses to the heart and maximum doses to the LAD (17.1 ± 13.1 Gy vs. 25.5 ± 16.0 Gy; *p* < 0.001). Coronary disease, including reduced perfusion, mainly occurs more than 10 years after irradiation [8, 25, 28]. Therefore, the LAD might be a substructure for dose reduction. However, due to its small volume, decreased visibility, and resultant contouring uncertainties in CT scans [29], it remains unclear whether it is superior to the mean dose of the heart or any other substructure.

Taken together, the model can identify patients who receive lower heart doses through application of DIBH; however, it warrants validation in an independent dataset. The extent of the clinical benefit for patients cannot be concluded from our study, as this would require a much larger patient cohort with long-term clinical outcomes. Further limitations are the retrospective monocentric nature of the study, missing randomization, and exclusion of patients for DIBH planning CT scans with the mean heart dose up to 5 Gy criterion already met (according to the internal SOP relevant at the time). The latter also adds to the bias of the preselection of patients for DIBH planning CT, together with other clinical parameters potentially affecting the clinician’s decision, such as application of chemotherapy. In addition, one cannot rule out that patients with a mean heart dose of less than 5 Gy might also develop chronic heart disease after irradiation and, thus, also benefit from DIBH. Also, patients with lymph node irradiation other than in axilla levels I and II were not considered. In conclusion, the model is easy to apply and may potentially guide the clinician’s decision for the individual patient receiving radiotherapy of the left breast regarding the prescription of a DIBH planning CT, thus also saving time in the planning process. Future investigations including outcome data are needed to prove the patient benefit in terms of reduction of chronic heart toxicity through application of DIBH irradiation.
